# Development and validation of the conversational AI dependence scale for Chinese college students

**DOI:** 10.3389/fpsyg.2025.1621540

**Published:** 2025-07-31

**Authors:** Yuanyuan Chen, Mengyun Wang, Shujuan Yuan, Yan Zhao

**Affiliations:** ^1^Department of Psychology, Shaanxi University of Technology, Hanzhong, China; ^2^Department of Psychology, Anhui University of Chinese Medicine, Hefei, China

**Keywords:** conversational artificial intelligence dependence, validation, scale, psychometric, Chinese college students

## Abstract

Excessive dependence on Conversational artificial intelligence (CAI) can significantly impact individual adaptation and development. Given the growing need for empirical assessment, this study presents the development and psychometric validation of the CAI Dependence Scale (CAIDS), a new instrument designed to assess CAI dependence among Chinese college students. In Study 1, drawing on theories of problematic internet use (PIU) and qualitative interviews, we identified the psychological connotations and dimensions of CAI dependence. Item and exploratory factor analyses led to the development of the 20-item CAIDS, comprising four dimensions: uncontrollability, withdrawal symptoms, mood modification, and negative impacts. In Study 2, confirmatory factor analysis in a new sample validated the four-dimensional structure and demonstrated good reliability and validity. In Study 3, a current status survey revealed that the overall level of CAI dependence among college students was relatively high, with significant differences observed by gender, age, grade, income, and region. CAI dependence was a significant positive predictor of negative psychological outcomes and a significant negative predictor of subjective wellbeing. Withdrawal symptoms and negative impacts were more closely related to maladaptive indicators. The CAIDS provides a reliable and valid psychometric tool for assessing CAI dependence; additionally, further validation is required with more diverse samples and in cross-cultural contexts.

## 1 Introduction

Conversational artificial intelligence (CAI) technologies refer to intelligent behaviors manifested through language interactions such as conversations and question answering, as exhibited by Chatbot's and intelligent assistants. CAI is one of the most challenging and pervasive areas of AI. Because of its interactivity, convenience, and highly personalized services, CAI has become increasingly indispensable in people's daily lives (Khatri et al., 2018). By December 2024, the adoption rate of AI in China, as represented by large language models, had reached 17.7% (CNNIC, 2025). Surveys indicate that AI awareness among adolescents is remarkably high, with 45.1% of minors using AI products (Fang et al., 2024). Additionally, over 80% of Chinese college students surveyed reported using AI tools (Bi et al., 2023). These findings suggest that the use of AI tools is significantly skewed toward younger users, with Millennials and Generation Z serving as the primary users of AI technologies. However, whether AI is beneficial or disadvantageous for humanity remains a contentious issue (Wei et al., 2024). Research indicates that increased interaction frequency between users and CAI may foster psychological attachment, thereby intensifying addictive usage and compulsive dependency (Hu et al., 2023; Ramadan, 2021). Such dependency on CAI, though similarly compulsive in nature, diverges substantially from users' reliance on other internet applications. Unlike the intermediary roles of smartphones or social media, CAI engages users directly through verbal or text-based interactions (Guzman and Lewis, 2020). Additionally, CAI usage is inherently dynamic, involving multi-turn conversations, which contrasts with the passive and aimless activities often associated with internet or social media use, such as browsing, reading, and watching (Frison and Eggermont, 2020; Skantze, 2021). Finally, CAI usage may lead to a more isolated experience, focusing on one-on-one interactions with AI agents rather than the group engagement typical of social media (Hu et al., 2023). Considering these unique distinctions, it is necessary to extend the research scope of problematic internet use (PIU) to encompass CAI.

PIU is considered a broad, umbrella term rather than a single diagnostic category (Fineberg et al., 2022). It encompasses a range of maladaptive online behaviors, including internet gaming disorder, problematic smartphone use, compulsive online shopping, and social media addiction (Brand, 2022). In addition, numerous theoretical models have emerged to explain the development and maintenance of PIU. Several frameworks offer valuable insights into these behaviors, including the compensatory internet use theory, uses and gratifications theory, the cognitive-behavioral model of pathological internet use, and the Interaction of Person-Affect-Cognition-Execution (I-PACE) model (Brand et al., 2019; Busch and McCarthy, 2021; Elhai et al., 2019; Kardefelt-Winther, 2014). In recent years, building on an in-depth understanding of PIU, as well as the aforementioned theories and empirical studies, researchers in China have recently adapted several measurement tools to assess compulsive behaviors associated with excessive dependence on CAI. Representative tools include the Problematic CAI Use Scale (Hu et al., 2023), adapted from the Bergen Social Media Addiction Scale (BSMAS; Andreassen et al., 2016), which assesses six dimensions of behavioral addiction: salience, mood modification, tolerance, withdrawal, conflict, and relapse; and the Compulsive Social Chatbot Conversation Scale (Ali et al., 2024), a unidimensional instrument derived from the compulsive smartphone usage research by Panda and Jain (2018). Although previous studies have established the reliability and validity of these tools, such tools have notable shortcomings. First, these tools predominantly use simplified, unidimensional item structures, relying solely on total scores to indicate dependency levels, lacking sufficient attention and in-depth examination of the different dimensions of dependency. Second, commonly used measurement tools were primarily adapted from social media and smartphone addiction scales, which partially overlook the unique characteristics of CAI dependency, such as cognitive outsourcing and impaired social functioning, focusing primarily on commonalities with PIU while lacking exploration of the unique features of CAI (Davidson et al., 2022). Third, the applicability of the adapted tools in the Chinese cultural context requires further investigation and validation.

Previous research examining the relationship between humans and CAI has largely focused on aspects such as usage intensity (Ng, 2024), usage types (Laestadius et al., 2024), compulsive use (Ali et al., 2024), and usage-related issues (Hu et al., 2023), while rarely considering a broader perspective. Moreover, the conceptual structure, characteristics, and current developments of CAI dependency remain inadequately explored. To better understand and assess CAI dependency, this study integrates qualitative research and psychometric methods to construct the psychological connotations and characteristic dimensions of this emerging social phenomenon in the Chinese context. Accordingly, the CAI Dependence Scale (CAIDS) for college students was developed, providing a theoretical basis and effective tool for future research. The three studies proceeded as follows: (1) Study 1 employed qualitative research to construct the psychological connotations and characteristic dimensions of CAI dependency and utilized quantitative methods to develop the CAIDS; (2) Study 2 further tested the reliability and validity of the CAIDS in a different sample; and (3) Study 3 conducted a preliminary survey among college students to evaluate the scale's effectiveness in practice and further explore the prevalence and developmental status of CAI dependency within this specific group.

## 2 Study 1: initial development of the CAIDS: theoretical construction and scale development

### 2.1 Theoretical construction

Drawing on the standards of the addiction component model proposed by Griffiths (2005) and research on PIU, including problematic CAI use (Hu et al., 2023), compulsive use of social Chatbot's (Ali et al., 2024), smartphone addiction (Kwon et al., 2013), the BSMAS (Andreassen et al., 2016), short-form video application addiction (Zhang et al., 2019), online gaming addiction (Charlton and Danforth, 2007; Pontes and Griffiths, 2015; Rehbein et al., 2015), and internet addiction (Caplan, 2010; Demetrovics et al., 2016; Meerkerk et al., 2009; Young, 1998), this study identified the core dimensions of CAI dependence. Following extensive discussions with the interview team, an interview outline was developed, resulting in an 11-item interview questionnaire (see Appendix A). The interview content was structured into two main sections: (1) the first section systematically collects demographic information (gender, age, major, grade, income, region) and background data on CAI use, including key indicators such as usage duration, frequency, functions used, and overall dependence level; (2) the second section, based on the interview outline, explores participants' motivations for using CAI, usage scenarios, psychological and behavioral characteristics, and the various impacts on individuals. To ensure the scientific rigor and validity of the outline, a pilot interview was conducted with four master's students. Based on their feedback, further revisions were made to finalize the interview framework.

Following approval from the Research Ethics Committee at the first author's institution, informed consent was obtained from all participants. A total of 64 college students from various majors, all with experience using CAI, were initially screened with the question: “To what extent do you feel dependent on CAI?” Responses were rated on a 9-point Likert scale, ranging from 1 (“not at all dependent”) to 9 (“completely dependent”), with higher scores indicating greater dependence. Ultimately, 31 students (16 female, 15 male; *M* = 20.87 years, *SD* = 1.54), whose scores exceeded the mean of 7.88, were selected for inclusion. These participants, reporting a high level of CAI dependence, were recruited from 18 provinces across China. The researchers conducted semi-structured interviews using the questionnaire and applied consensual qualitative research (Hill et al., 2005) to analyze the interview content in-depth. The study found that CAI dependence refers to a psychological and behavioral state in which individuals develop a persistent and intense need for CAI due to excessive use, leading to significant impairments in cognitive, emotional, behavioral, and social functioning. CAI dependence is a specific form of technology addiction, classified as a non-chemical behavioral dependency that involves human-computer interaction. The interviews revealed several key characteristics of CAI dependence, including behavioral features (salience, tolerance, withdrawal symptoms, and relapse), emotional features (mood modification and virtual intimacy), cognitive outsourcing (cognitive laziness and information processing dependence), and impaired social functioning (conflict and withdrawal).

### 2.2 Development of the CAIDS for college students

#### 2.2.1 Participants

A total of 620 college students with experience using CAI were recruited as initial test participants through convenience sampling from several cities, including Shanghai, Guangzhou, Xi'an, Haikou, and Hefei. All participants completed a preliminary version of the CAIDS. After excluding questionnaires with patterned responses or incomplete information, 547 valid responses were retained, representing 88.23% of the original sample. Participants ranged in age from 17 to 25 years (*M* = 20.59, *SD* = 1.66).

#### 2.2.2 Initial questionnaire development and research procedures

Based on a literature review, qualitative research findings, and questionnaires related to PIU—including the Problematic CAI Use Scale (Hu et al., 2023), the Compulsive Social Chatbot Use Scale (Ali et al., 2024), the Smartphone Addiction Scale (SAS; Kwon et al., 2013), the Bergen Social Media Addiction Scale (Andreassen et al., 2016), the Excessive Use of Short-Video Applications Scale (Zhang et al., 2023), the Internet Gaming Disorder Scale (IGDS-SF9; Pontes and Griffiths, 2015), and the Problematic Internet Use Questionnaire (PIUQ-SF-6; Demetrovics et al., 2016)—the preliminary version of the CAIDS for Chinese college students was developed. By integrating the content of these questionnaires and the qualitative interview results, eight dimensions of CAI dependence were identified. Following extensive discussions and multiple rounds of modification, addition, deletion, and adjustment by four PhD students specializing in basic psychology and two PhD students specializing in developmental and educational psychology, a final set of 51 initial items was established. The dimensions included salience (6 items), tolerance (6 items), withdrawal symptoms (8 items), relapse (8 items), mood modification (7 items), virtual intimacy (5 items), cognitive outsourcing (4 items), and social dysfunction (6 items). A six-point Likert scale was used, whereby participants rated their use of CAI, with 1 representing “completely disagree” and 6 representing “completely agree.” Higher scores indicated a higher level of dependency on CAI.

Following approval from the Research Ethics Committee at the first author's institution, informed consent was obtained from all participants, schools, and teachers. A questionnaire survey was then conducted through a combination of in-class and online group tests, with participation being voluntary and privacy protection emphasized. Participants were encouraged to answer truthfully based on their real feelings and to complete the questionnaire within the allotted time.

#### 2.2.3 Results

Exploratory factor analysis (EFA) was conducted using SPSS 22.0. The total scores of the initial 51-item questionnaire were calculated, with the top 27% of participants classified into the high-score group and the bottom 27% into the low-score group. The average scores for each item were compared between these groups to determine whether significant differences existed. If no significant difference was found, the item was considered to have low discrimination and was subsequently removed (Wang et al., 2022). The results indicated that all 51 items exhibited significant differences between the high- and low-scoring groups (*p* < 0.001), suggesting that the items in the initial questionnaire had good discrimination.

An EFA was conducted to validate the rationality of the proposed dimensions of the questionnaire and further examine the homogeneity between individual items, the overall questionnaire, and its dimensions. In this study, the Kaiser-Meyer-Olkin value of the initial questionnaire data was 0.95, and the chi-square value for Bartlett's test of sphericity was 8,619.67, with 190 degrees of freedom, *p* < 0.001. These results supported the need for further EFA. Principal components analysis was used for the EFA, with the factor loading matrix obtained through varimax rotation. Factors with eigenvalues >1 were extracted, and items with communalities < 0.4 were removed. After rotation, items with factor loadings < 0.4, those loading on multiple factors with values >0.4, and factors containing fewer than three items were deleted. This resulted in a four-factor model. The scree plot shows a cumulative variance explained of 74.41%. The final factor analysis results for the 20 remaining items are presented in Table 1.

**Table 1:** The four characteristic dimensions of the CAIDS.

**Item**	**Uncontrollability**	**Withdrawal symptoms**	**Mood modification**	**Negative impact**	**Communality**
33	0.79				0.72
34	0.78				0.78
36	0.74				0.73
47	0.68				0.63
25	0.66				0.67
9	0.65				0.68
11		0.82			0.84
14		0.80			0.77
12		0.79			0.83
15		0.73			0.73
27			0.81		0.83
28			0.78		0.81
30			0.71		0.77
29			0.70		0.75
44			0.59		0.59
4				0.77	0.76
1				0.74	0.74
3				0.73	0.76
5				0.70	0.70
6				0.66	0.69
Eigenvalues	3.95	3.70	3.69	3.53	
Contribution rate	19.75%	18.51%	18.48%	17.67%	
Accumulating contribution rate	19.75%	38.25%	56.73%	74.41%	

N = 547.

Based on the results of the EFA, the final version of the CAIDS consisted of 20 items divided into four factors: uncontrollability, withdrawal symptoms, mood modification, and negative impact. The first factor, uncontrollability, describes the degree to which individuals lack control over their use of CAI, including frequent usage and increased tolerance, and represents the most direct aspect of assessing AI dependency. The second factor, withdrawal symptoms, describes the unpleasant feelings experienced when users are unable to engage in CAI because of issues such as network problems, time restrictions, or server crashes. The third factor, mood modification, describes how the use of CAI helps improve user emotions, such as shifting from negative to positive states. The fourth factor, negative impact, describes the conflicts arising from AI dependency in relation to real-life social relationships or other activities as well as the retreat in social adaptation and competence.

## 3 Study 2: reliability analyses and measurement invariance tests

### 3.1 Participants

Following approval from the Research Ethics Committee at the first author's institution, informed consent was obtained from all participants. A total of 750 college students with prior experience using CAI were recruited through convenience sampling from Shanghai, Guangzhou, Xi'an, Haikou, and Hefei. All participants completed the final version of the CAIDS and the criterion scale. After excluding questionnaires with patterned responses or incomplete information, 687 valid responses were retained, representing 91.60% of the original sample. Participants ranged in age from 17 to 28 years (*M* = 20.41, *SD* = 1.63).

### 3.2 Measures

#### 3.2.1 The CAIDS-20

The CAIDS-20 as explained in Study 1.

#### 3.2.2 Social media addiction scale

This study employed the Bergen Social Media Addiction Scale (BSMAS) developed by Andreassen et al. (2016). The BSMAS comprises six items reflecting the six core components of addiction identified by Griffiths (2005): salience, mood modification, tolerance, withdrawal, conflict, and relapse. Example items include “I spend a lot of time thinking about or planning to use social media” and “I increasingly crave using social media.” Responses are rated on a five-point Likert scale ranging from 1 (very rarely) to 5 (very often), with higher total scores indicating greater levels of addiction. The BSMAS has been widely used among Chinese college students and has demonstrated good reliability and validity (Lu et al., 2025). The Cronbach's alpha in this study was 0.84.

#### 3.2.3 Short version of the loneliness scale

This study employed the short version of the University of California, Los Angeles (UCLA) Loneliness Scale developed by Hughes et al. (2004). The scale consists of three items that assess how frequently individuals experience feelings of lacking companionship, being left out, and feeling isolated from others. Example items include, “How often do you feel that you lack companionship?” and “How often do you feel left out?” Responses are rated on a five-point Likert scale ranging from 1 (never) to 5 (always), with higher total scores indicating greater levels of loneliness. The validity of this scale has been confirmed in adult Chinese populations (Hu et al., 2023; Liu et al., 2020). The Cronbach's alpha in this study was 0.85.

#### 3.2.4 Short version of the experiences in close relationships scale

This study employed the short version of the Experiences in Close Relationships Scale (ECR-M16) developed by Lo et al. (2009). This scale consists of 16 items measuring two dimensions: attachment anxiety and attachment avoidance. Example items include “I need a lot of reassurance that those I feel close to love me” and “I am uncomfortable opening up to others.” Responses are rated on a seven-point Likert scale ranging from 1 (strongly disagree) to 7 (strongly agree), with higher total scores indicating greater attachment insecurity. The Cronbach's alpha in this study was 0.87.

### 3.3 Results

#### 3.3.1 Structural validity analysis

Confirmatory factor analysis (CFA) was conducted using Mplus 8.3 to evaluate the fit of the four-factor model of CAI dependence based on a formal questionnaire. The model fit indices were χ^2^/*df* = 4.39, RMSEA = 0.07, SRMR = 0.06, CFI = 0.92, and TLI = 0.91. The CFA results indicated that the sample data fit the model reasonably well, supporting the four-factor model structure.

#### 3.3.2 Convergent validity

The factor loadings for the items corresponding to the four dimensions of CAI dependence were all >0.5, indicating that they were highly representative of their respective dimensions. Additionally, the average variance extracted (AVE) for each dimension ranged from 0.55 to 0.72 (>0.4) and the composite reliability ranged from 0.86 to 0.91 (>0.8), indicating that the convergent validity of the model was good.

#### 3.3.3 Discriminant validity

Discriminant validity was tested for four dimensions of CAI dependence. The results (Table 2) indicated that the correlations between the four dimensions of CAI dependence were all significant and the correlation coefficients were all less than the square root of the corresponding AVE. This suggests that the dimensions were somewhat correlated while maintaining distinctiveness, indicating that the questionnaire has good discriminant validity.

**Table 2:** Discriminant validity of each dimension of the CAIDS.

**Dimension**	**1**	**2**	**3**	**4**
1. Uncontrollability	–			
2. Withdrawal symptoms	0.60^***^	–		
3. Mood modification	0.65^***^	0.50^***^	–	
4. Negative impact	0.61^***^	0.77^***^	0.59^***^	–
The square root of AVE	0.74	0.83	0.85	0.75

N = 687. ^***^p < 0.001.

#### 3.3.4 Reliability analysis

The internal consistency and split-half reliability coefficients for the total score and each dimension (uncontrollability, withdrawal symptoms, mood modification, and negative impact) of the formal questionnaire were examined. The results showed that the internal consistency coefficients for the total score and each dimension were 0.86, 0.91, 0.91, 0.88, and 0.94, respectively, while the split-half reliability coefficients were 0.81, 0.86, 0.90, 0.88, and 0.77, respectively.

#### 3.3.5 Criterion-related validity

Previous research has shown that PIU is often associated with higher levels of social media addiction, loneliness, and insecure attachment (Barreto Carvalho et al., 2023; Ergün et al., 2023; Hu et al., 2023; Yildirim Demirdögen et al., 2024). In this study, social media addiction, loneliness, and attachment style scales were used as criteria for the CAIDS. As shown in Table 3, the CAIDS and its four dimensions were significantly and positively correlated with years of use, usage frequency, usage duration, social media addiction, loneliness, and attachment style.

**Table 3:** Criterion-related validity analysis.

**Item**	**1**	**2**	**3**	**4**	**5**	**6**	**7**	**8**	**9**	**10**	**11**
1. Years of use	–										
2. Usage frequency	0.39^***^	–									
3. Usage duration	0.45^***^	0.39^***^	–								
4. Uncontrollability	0.28^***^	0.44^***^	0.26^***^	–							
5. Withdrawal symptoms	0.22^***^	0.20^***^	0.21^***^	0.55^***^	–						
6. Mood modification	0.36^***^	0.39^***^	0.26^***^	0.59^***^	0.47^***^	–					
7. Negative impact	0.22^***^	0.21^***^	0.26^***^	0.55^***^	0.74^***^	0.49^***^	–				
8. CAID	0.32^***^	0.36^***^	0.30^***^	0.79^***^	0.86^***^	0.75^***^	0.88^***^	–			
9. Social media addiction	0.08^*^	0.36	0.12^**^	0.30^***^	0.52^***^	0.26^***^	0.53^***^	0.51^***^	–		
10. Loneliness	0.07	−0.04	0.12^**^	0.13^***^	0.32^***^	0.15^***^	0.46^***^	0.35^***^	0.42^***^	–	
11. Insecure attachment	0.02	−0.08	0.05	0.11^**^	0.44^***^	0.10^*^	0.53^***^	0.39^***^	0.47^***^	0.64^***^	–
*M*	2.06	2.55	2.05	4.26	3.80	3.96	3.41	3.83	3.42	2.08	3.95
*SD*	1.10	1.10	1.30	.99	1.26	1.31	1.19	0.98	0.81	0.77	0.99

N = 687. ^*^p < 0.05; ^**^p < 0.01; ^***^p < 0.001.

## 4 Study 3: development status survey

Studies 1 and 2 systematically examined the conceptual connotations and structural characteristics of CAI dependence by combining qualitative and quantitative research methods. In Study 3, we built on the findings of previous studies to further investigate the prevalence and developmental level of CAI dependence among college students. Specifically, this study explored the general aspects of CAI dependence, considering demographic factors (such as gender, age, grade, income, and region) as well as aspects related to physiological, psychological, and social adaptation (including sleep issues, functional difficulties, depression, anxiety, stress, and subjective wellbeing). This study thoroughly explored the impact of CAI dependence and its dimensions on these indicators, providing preliminary empirical evidence for future research.

### 4.1 Participants

Following approval from the Research Ethics Committee at the first author's institution, informed consent was obtained from all participants. The study involved two independent samples. The study involved two independent samples. Sample 1 was used to investigate the general status of CAI dependence among college students, while Sample 2 focused on the relationship between CAI dependence and key physiological, psychological, and social adaptation indicators.

Sample 1. Participants with prior experience using CAI were recruited using convenience sampling from several universities in Xi'an, Haikou, Shanghai, Guangzhou, and Hefei. A total of 1,200 questionnaires were distributed to entire classes. After excluding questionnaires with patterned responses or incomplete information, 1,081 valid responses were retained (629 female students, 58.20%), yielding an effective response rate of 90.08%. The sample comprised 287 freshmen (26.50%), 335 sophomores (31.00%), 291 juniors (26.90%), 135 seniors (12.50%), and 33 graduate students (3.10%). Participants' ages ranged from 17 to 26 years (*M* = 20.23, *SD* = 1.73).

Sample 2. A total of 950 college students with prior experience using CAI were recruited through convenience cluster sampling from Xi'an and Haikou. After excluding questionnaires with patterned responses or incomplete information, 892 valid questionnaires were retained (526 female students, 59.00%), yielding an effective response rate of 93.89%. The sample consisted of 275 freshmen (30.80%), 306 sophomores (34.30%), 216 juniors (24.20%), 72 seniors (8.10%), and 23 graduate students (2.60%). Participants' ages ranged from 17 to 25 years (*M* = 19.97, *SD* = 1.63).

### 4.2 Measures

#### 4.2.1 Athens insomnia scale

The Athens Insomnia Scale, developed by the Ohio State University College of Medicine in 1985, was used to assess sleep quality. It is a widely recognized international measurement tool that includes eight items covering aspects such as time to fall asleep, frequency of awakening during the night, early morning awakening, total sleep duration, sleep quality, daytime mood, daytime physical functioning, and daytime sleepiness. Each item is rated on a 4-point Likert scale ranging from 0 (no problem) to 3 (severe problem), with higher total scores indicating poorer sleep quality. The Cronbach's alpha in this study was 0.83.

#### 4.2.2 Functional difficulties scale

This study employed the Functional Difficulties Scale developed by Tokunaga (2016), which consists of nine items covering three dimensions: friendship difficulties (e.g., “I have noticed that my connection with offline friends has decreased”), family difficulties (e.g., “I feel that my relationship with family members has become distant”), and academic difficulties (e.g., “I feel like I've been facing some difficulties in my studies recently”). Each item is rated on a seven-point Likert scale ranging from 1 (strongly disagree) to 7 (strongly agree), with higher total scores reflecting greater functional difficulties. In this study, Cronbach's alpha for the overall scale and the three subscales were 0.96, 0.95, 0.96, and 0.93, respectively.

#### 4.2.3 Depression-anxiety-stress scale

This study employed the Chinese version of the Depression-Anxiety-Stress Scale (DASS-21; Antony et al., 1998; Lovibond and Lovibond, 1995), revised by Gong et al. (2010). This scale consists of 21 items that assess symptoms of depression (e.g., “I feel that life is meaningless”), anxiety (e.g., “I felt scared without any good reason”), and stress (e.g., “I found it hard to tolerate interruptions to what I was doing”). Each item is rated on a four-point Likert scale ranging from 1 (never) to 4 (almost always), with higher total scores indicating more severe negative emotional states. In this study, Cronbach's alpha for the overall scale and for the depression, anxiety, and stress subscales were 0.96, 0.92, 0.92, and 0.91, respectively.

#### 4.2.4 Subjective wellbeing scale

This study employed the Ultra-Short Protocol for Measuring Subjective Wellbeing (USP-SWB; Skevington et al., 2004) to assess general subjective wellbeing. This scale comprises six items covering three key domains of the World Health Organization Quality of Life Scale: physical (overall health and sleep quality), psychological (life satisfaction and sense of meaning in life), and social (relationship satisfaction and satisfaction with support from friends). Each item is rated on a nine-point Likert scale ranging from 1 (very dissatisfied) to 9 (very satisfied), with higher total scores reflecting greater subjective wellbeing. This scale has demonstrated good validity and reliability in previous research (Balcerowska et al., 2022). The Cronbach's alpha in this study was 0.92.

#### 4.2.5 The CAIDS-20

As in Study 1. In both Samples 1 and 2, the CAIDS-20 demonstrated strong reliability and validity, with a Cronbach's alpha of 0.96.

### 4.3 Procedure

After obtaining informed consent from schools, teachers, and participants, a questionnaire survey was conducted using a combination of in-class and online group tests. Participation was voluntary, and privacy protection was ensured. Students were encouraged to respond honestly based on their actual feelings and were asked to complete the questionnaire within the allotted timeframe.

### 4.4 Statistical analysis

In this study, Harman's single-factor test was used to assess common method bias before conducting further data analysis. An unrotated principal components factor analysis was conducted for all of the survey items. The results showed that in Sample 1, eight factors had eigenvalues >1, cumulatively explaining 67.72% of the variance, with the first factor explaining 37.39% of the variance, which did not reach the standard threshold of 40%. In Sample 2, 13 factors had eigenvalues >1, cumulatively explaining 68.70% of the variance, with the first factor explaining 33.36% of the variance, which did not reach the standard threshold of 40%. These findings indicate that common method bias was not present in this study.

Subsequently, a descriptive statistical analysis was conducted to examine the basic characteristics of CAI dependency. Independent samples *t*-tests and analysis of variance (ANOVA) were used to explore differences in CAI dependence across different demographic variables (Sample 1). Correlation and path analyses were then employed to explore the relationship between CAI dependence and physiological, psychological, and social adaptability indicators (Sample 2).

### 4.5 Results

#### 4.5.1 Basic survey of CAI dependence

The survey on the general status of CAI dependence among college students found that the average number of years that students had used CAI was 1.91. More than 50% of college students spent more than 2 h per day using CAI, and over 74.8% used CAI almost every day. Regarding the functions used, the students primarily used CAI for virtual assistance and daily communication, answering questions and information retrieval, writing and creative generation, language translation and communication, and learning and educational support.

An independent samples *t*-test was used to examine gender differences in CAI dependency. The results indicated significant gender differences, with male students showing a significantly higher overall level of CAI dependency (*t* = 6.35, *p* < 0.001) and higher scores on all dimensions than female students (*t*_*Uncontrollability*_ = 6.90, *p* < 0.001; *t*_*WithdrawalSymptoms*_ = 3.19, *p* < 0.001; *t*_*Moodmodification*_ = 7.90, *p* < 0.001; *t*_*Negativeimpact*_= 4.80, *p* < 0.001).

One-way ANOVA was used to examine age differences in CAI dependency. The results indicated significant age differences, with older college students having a significantly higher overall level of CAI dependency (*F* = 26.33, *p* < 0.001) and higher scores on all dimensions than younger students (*F* = 27.96, *p* < 0.001; *F* = 15.11, *p* < 0.001; *F* = 33.13, *p* < 0.001; *F* = 10.57, *p* < 0.001). However, as age increased from higher (22 years) to the highest (26 years), CAI dependency showed a declining trend.

ANOVA was used to examine grade-level differences in CAI dependence. The results indicated significant grade-level differences (*F* = 44.12, *p* < 0.001). Furthermore, multiple comparisons showed that third- and fourth-year students had the highest levels of CAI dependency, with no significant differences between the two groups. This was followed by second-year and graduate students, whereas first-year students had the lowest levels of CAI dependency.

One-way ANOVA was used to examine differences in CAI dependency based on grade level. Annual family income was divided into 12 categories, ranging from “below 20,000 yuan” to “above 800,000 yuan,” and scores were assigned from 1 to 12 accordingly. The results indicated significant differences in CAI dependency based on annual family income (*F* = 12.21, *p* < 0.001). Further multiple comparisons revealed that college students from lower-income families had lower levels of CAI dependency, whereas students from middle-to high-income families had higher levels of dependency.

An independent samples *t*-test was conducted to examine differences in CAI dependency based on habitual residence. The results indicated significant rural-urban differences in the negative impact dimension of CAI dependency, with rural college students scoring significantly higher than their urban counterparts (*t* = 2.80, *p* < 0.01). However, no significant differences were found in the total CAI dependency score or the dimensions of uncontrollability, withdrawal symptoms, or mood modification.

#### 4.5.2 Correlation and regression analysis

To further explore the relationship between CAI dependence and indicators of physiological, psychological, and social adaptation, correlations were examined between college students' CAI dependence (Sample 2) and eight key indicators: sleep problems, functional difficulties (in friendships, family, and academics), depression, anxiety, stress, and subjective wellbeing. The results (Table 4) indicated that the total score of CAIDS and its dimensions were significantly positively correlated with negative indicators such as sleep problems, functional difficulties, depression, anxiety, and stress. Withdrawal symptoms and negative impact were significantly negatively correlated with subjective wellbeing.

**Table 4:** Descriptive statistics and correlations.

**Variable**	**Sleep problems**	**Friendship difficulties**	**Family difficulties**	**Academic difficulties**	**Depression**	**Anxiety**	**Stress**	**Subjective wellbeing**
Uncontrollability	0.24^***^	0.55^***^	0.54^***^	0.44^***^	0.25^***^	0.33^***^	0.26^***^	0.06
Withdrawal symptoms	0.35^***^	0.64^***^	0.63^***^	0.59^***^	0.41^***^	0.46^***^	0.44^***^	−0.11^**^
Mood modification	0.17^***^	0.60^***^	0.59^***^	0.41^***^	0.27^***^	0.33^***^	0.25^***^	0.05
Negative impact	0.45^***^	0.78^***^	0.79^***^	0.71^***^	0.54^***^	0.57^***^	0.50^***^	−0.15^***^
CAID	0.35^***^	0.74^***^	0.73^***^	0.62^***^	0.43^***^	0.49^***^	0.42^***^	−0.05
*M*	2.11	3.05	2.91	3.69	1.87	1.72	1.86	5.20
*SD*	0.66	1.78	1.82	1.75	0.73	0.71	0.74	1.25

N = 892. ^**^p < 0.01; ^***^p < 0.001.

Controlling for gender, age, grade level, and usage intensity (duration, frequency, and daily usage time), the CAID was treated as the independent variable, while the eight key indicators were considered dependent variables. This approach was used to examine the predictive role of CAID on these physiological, psychological, and social adaptation indicators. The results presented in Table 5 demonstrate that the CAIDS, along with its four dimensions, significantly positively predicted increased levels of negative outcomes, including sleep problems, functional difficulties, depression, anxiety, and stress. Moreover, the total score of CAIDS, withdrawal symptoms, and negative impact dimensions were significantly negatively predicted to subjective wellbeing.

**Table 5:** Regression analysis.

**Regression equation**		**Overall fit index**			**Regression equation**		**Overall fit index**		
**Outcome variable**	**Predictor variable**	***R** ^2^*	β	***S.E***.	**Outcome variable**	**Predictor variable**	***R** ^2^*	β	***S.E***.
Sleep problems	Uncontrollability	0.09^***^	0.28^***^	0.02	Depression	Uncontrollability	0.10^***^	0.27^***^	0.02
	Withdrawal symptoms	0.16^***^	0.39^***^	0.02		Withdrawal symptoms	0.21^***^	0.45^***^	0.02
	Mood modification	0.06^***^	0.16^***^	0.02		Mood modification	0.10^***^	0.28^***^	0.02
	Negative Impact	0.23^***^	0.50^***^	0.02		Negative impact	0.33^***^	0.60^***^	0.02
	CAID	0.17^***^	0.45^***^	0.02		CAID	0.24^***^	0.53^***^	0.02
Friendship difficulties	Uncontrollability	0.35^***^	0.46^***^	0.05	Anxiety	Uncontrollability	0.14^***^	0.34^***^	0.02
	Withdrawal symptoms	0.46^***^	0.56^***^	0.03		Withdrawal symptoms	0.24^***^	0.47^***^	0.02
	Mood modification	0.39^***^	0.53^***^	0.04		Mood modification	0.13^***^	0.31^***^	0.02
	Negative impact	0.62^***^	0.73^***^	0.03		Negative impact	0.33^***^	0.60^***^	0.02
	CAID	0.56^***^	0.74^***^	0.04		CAID	0.28^***^	0.56^***^	0.02
Family difficulties	Uncontrollability	0.34^***^	0.45^***^	0.05	Stress	Uncontrollability	0.09^***^	0.29^***^	0.02
	Withdrawal Symptoms	0.45^***^	0.55^***^	0.04		Withdrawal symptoms	0.21^***^	0.48^***^	0.02
	Mood modification	0.38^***^	0.52^***^	0.04		Mood modification	0.07^***^	0.25^***^	0.02
	Negative impact	0.63^***^	0.74^***^	0.03		Negative impact	0.27^***^	0.55^***^	0.02
	CAID	0.55^***^	0.73^***^	0.04		CAID	0.21^***^	0.52^***^	0.02
Academic difficulties	Uncontrollability	0.21^***^	0.44^***^	0.05	Subjective wellbeing	Uncontrollability	0.02	0.02	0.04
	Withdrawal symptoms	0.35^***^	0.58^***^	0.04		Withdrawal symptoms	0.04^***^	−0.17^***^	0.03
	Mood modification	0.18^***^	0.39^***^	0.04		Mood modification	0.02	0.01	0.03
	Negative impact	0.51^***^	0.74^***^	0.04		Negative impact	0.06^***^	−0.22^***^	0.04
	CAID	0.40^***^	0.70^***^	0.05		CAID	0.40^**^	−0.14^**^	0.04

N = 892. ^**^p < 0.01; ^***^p < 0.001.

## 5 Discussion

The CAIDS-20 for college students was developed in three stages. Using qualitative interviews and theories related to PIU, we explored the psychological constructs and dimensions of CAI dependence. Through item and exploratory factor analyses, a 20-item scale comprising four dimensions—uncontrollability, withdrawal symptoms, mood modification, and negative impact—was established. In the second stage, CFA was employed to validate these four dimensions with a new sample, demonstrating that the CAIDS-20 has good reliability and validity. Finally, an extensive survey of college students was conducted to verify the efficacy of the scale in practical applications, providing an in-depth understanding of the prevalence and development of CAI dependence within this specific population.

Qualitative research provided an initial understanding of CAI dependence, clarifying its conceptual meaning and multidimensional characteristics. The findings of the qualitative study align with previous research on PIU, such as that on social media and smartphone addiction (Andreassen et al., 2016; Elhai et al., 2020; Montag et al., 2021; Stănculescu and Griffiths, 2022). From one perspective, both CAI dependence and PIU exhibit behavioral features such as salience, tolerance, withdrawal symptoms, and relapse. Excessive use of these technologies may result in difficulties in daily life, such as impaired performance in social, academic, and work contexts (Hu et al., 2023; León-Domínguez, 2024). Further, both forms of dependence involve the use of technology as an emotional regulation tool. Whether through virtual intimacy established via social media or emotional support gained through AI interaction, users tend to seek technology to fulfill their emotional needs (Laestadius et al., 2024). However, CAI dependence differs from generalized PIU in several respects. While internet addiction also relies on information processing, CAI dependence may be more pronounced in terms of cognitive outsourcing, whereby users delegate tasks that would otherwise require their own thinking and decision-making to AI tools, consequently reducing their cognitive effort (Royer, 2024). However, excessive dependence can lead to cognitive inertia, extending beyond traditional online content consumption (Skulmowski, 2023). Moreover, CAI offers a more personalized and dynamic form of interaction that can intensify user dependence. By contrast, problematic traditional internet use is largely characterized by content consumption and static interactions. Interactivity with AI fosters stronger emotional connections and higher levels of emotional dependence.

Through item and exploratory factor analyses, CAI dependence was conceptualized into four core dimensions: uncontrollability, withdrawal symptoms, mood modification, and negative impact. Subsequently, CFA was conducted using a new sample to assess the reliability and validity of the CAIDS. The results demonstrated good reliability and validity, confirming the robustness of its structure. Notably, the “uncontrollability” dimension (comprising five items) integrates the features of salience and tolerance revealed in the qualitative interviews, drawing on the approach used by Chinese researchers to combine these two elements into a unified model when investigating problematic social media use (Peng and Liao, 2023). A recent study by Fournier et al. (2023) reported similar findings, suggesting that salience and tolerance are peripheral rather than core components of problematic social media use. Leung (2008) developed a Mobile Dependence Index based on the DSM-IV criteria for addiction, in which the uncontrollability dimension reflects an individual's inability to manage excessive time spent on the phone, encompassing both physical overuse and psychological craving, which together manifest the characteristics of “uncontrollability.” Additionally, the “negative impact” dimension (six items) combines conflict and cognitive inertia, as identified in the qualitative interviews, describing how CAI dependence can negatively impact daily life, work, academic performance, and interpersonal relationships, and potentially impair cognitive function.

Study 3 conducted an in-depth investigation into the prevalence and progression of CAI dependence among contemporary college students, revealing that, on average, the students had used CAI for 1.91 years. Over 50% of the college students spent more than 2 h per day interacting with AI, and over 74.8% used CAI almost every day, which is consistent with a recent survey by the China Youth Daily, indicating widespread overreliance on CAI among students (Bi et al., 2023). Specifically, college students primarily use CAI for functions such as virtual assistance, daily communication, question and answer-based information retrieval, content generation, language translation, and educational support. These findings suggest that CAI has become deeply embedded in students' daily lives, characterized by frequent and prolonged usage. This aligns with previous research, indicating the widespread application of CAI by students across various domains, including social interaction, entertainment, learning, and work (Guzman, 2018; Miner et al., 2020). The study also identified significant gender differences in CAI dependence, with male students exhibiting higher overall dependence levels and scores across all dimensions than female students. This disparity may be attributed to different usage motivations: women are more inclined to use CAI to maintain interpersonal relationships and emotional communication, whereas men may have a greater preference for systematic thinking, making them more susceptible to the novelty and complexity of technology, thereby increasing the risk of dependence (Jin and Eastin, 2024). Male users are more likely to develop dependence if they perceive increased efficiency and satisfaction when using CAI. By contrast, female users may reduce their usage frequency and dependence if they are dissatisfied with the utility and personalization of CAI (Liu and Yao, 2023).

The study found that income disparities significantly influence CAI dependency. Participants from lower-income families demonstrated lower levels of dependency, while those from middle- to high-income families exhibited greater reliance. This disparity is likely due to factors such as technology access, social norms, and educational support. Additionally, significant urban-rural differences were found only in the dimension of negative impact, with rural college students scoring significantly higher than their urban peers. These findings underscore the critical role of economic conditions and the urban-rural context in shaping technology-dependent behaviors, emphasizing the need to consider these factors when developing intervention strategies and educational guidance (Chen and Huo, 2023). Finally, this study demonstrated that CAI dependence significantly predicts negative physiological, psychological, and social adaptation indicators, including sleep problems, functional difficulties (i.e., friendship, family, and academic difficulties), depression, anxiety, and stress, while also being a significant negative predictor of subjective wellbeing. Excessive use of CAI can lead to attentional issues and negatively affect sleep quality. For instance, using CAI devices at night may disrupt the sleep cycle owing to blue light exposure, resulting in sleep disturbances. Previous studies demonstrated a negative correlation between screen time and sleep quality (Santos et al., 2023). Moreover, reliance on CAI may reduce face-to-face social interactions by substituting them with interactions via AI agents, which can impair real-world relationships and lead to difficulties in friendships and family functioning. This “social substitution effect” may weaken individuals' real-world social support networks, heightening loneliness, and social anxiety (Crawford et al., 2024). Additionally, research has shown that excessive reliance on CAI for information retrieval and learning tasks weakens deep learning and critical thinking skills, thereby contributing to academic challenges (van den Berg and du Plessis, 2023). Furthermore, CAI dependence may serve as an unhealthy emotion-regulation strategy, leading to increased levels of depression, anxiety, and stress. Individuals may use CAI to escape real-life pressures and negative emotions, but its excessive use may ultimately increase the risk of mental health issues (Hu et al., 2023). In summary, excessive reliance on CAI negatively affects physiological, psychological, and social adaptation, leading to a decline in subjective wellbeing. In other words, when individuals' life conditions deteriorate because of AI dependence, their overall satisfaction and wellbeing also diminish (Qu et al., 2023; Twenge and Campbell, 2018). The CAIDS-20 also has several practical application scenarios. For example, in the realm of mental health, the scale could serve as a crucial tool for identifying college students at risk of excessive dependency on CAI, facilitating early interventions and personalized support. Additionally, its integration into AI product design could guide the development of more user-centered, adaptive AI systems that account for the psychological effects of technology use, promoting healthier engagement patterns.

### 5.1 Limitations and directions for future research

These findings confirm prior work but have certain limitations. First, while the cross-sectional design offers valuable theoretical insights, it limits the ability to make causal inferences. Future research could employ experimental or longitudinal designs to more robustly examine the directionality of the observed relationships. Second, at the thematic level, this study systematically examined the conceptual structure and characteristics of generalized CAI dependence, but did not address the unique features of specific subtypes of CAI use (e.g., academic vs. social applications). Future research could further investigate the distinctions among generalized CAI dependence, dependence on particular AI subtypes, and other forms of PIU. Third, in terms of methodology, this study combined qualitative methods with quantitative scales but did not delve deeper into CAI dependence from a neuroimaging perspective. Future research should incorporate techniques such as brain imaging and eye tracking to provide neurocognitive validation. Fourth, this study primarily utilized a sample of Chinese undergraduate and graduate students. While this group demonstrates a high level of technological acceptance and widespread use of CAI applications, the demographic homogeneity may limit the generalizability of our findings. Future studies should include additional groups, such as adolescents and working adults, and seek to replicate and extend these findings using more heterogeneous samples from diverse cultural, educational, and demographic backgrounds.

### 5.2 Conclusion

The CAIDS-20 for Chinese college students consists of four dimensions: uncontrollability, withdrawal symptoms, mood modifications, and negative impacts. The CAIDS-20 scale developed in this study is an accessible, reliable, and valid tool for assessing psychological traits associated with CAI dependence. The findings revealed that the overall level of CAI dependence among Chinese college students is relatively high, with significant differences observed in terms of gender, age, academic year, income, and urban-rural residence. CAI dependence was a significant positive predictor of negative indicators related to physiological, psychological, and social adaptation and a significant negative predictor of subjective wellbeing. Notably, withdrawal symptoms and their negative impacts exhibited a stronger relationship with these adaptation indicators. This study provides reliable insights that serve as a foundation for advancing research on CAI dependence.

## Data Availability

The data analyzed in this study is subject to the following licenses/restrictions: Data available from corresponding author upon request. Requests to access these datasets should be directed to Yuanyuan Chen, chenyuanyuan@snut.edu.cn.

## Appendix A

**Qualitative research interview outline on CAI dependence**
Q1. How is your experience with using CAI? Why do you use it?
Q2. What aspects of CAI attract you? Compared to social apps, what points make it more appealing to you?
Q3. In what scenarios or under what needs do you use CAI? Why?
Q4. How do you think using CAI helps you?
Q5. When you encounter troubling issues, who do you first turn to for advice? Do you feel that CAI understands you better than they (friends or family)? Why?
Q6. Do you often use CAI without realizing it? Why?
Q7. Have you used CAI more frequently than when you first started? Has the time spent on it increased? Why?
Q8. Have you tried to reduce or stop using CAI? What was the outcome?
Q9. When you cannot access CAI, do you feel anxious? Are there other emotional responses besides anxiety?
Q10. When you encounter a problem, do you first consider asking CAI for help to solve it or make decisions? Why?
Q11. Has using CAI affected your ability to think independently? (Are there other effects?) Why?

## Appendix B

**Conversational artificial intelligence dependence scale (CAIDS-20)**
*In the Past Year..*.
I often spend a lot of time thinking about or planning to use CAI.
CAI has become an indispensable part of my daily life and studies.
I find myself using CAI more and more frequently.
Each time I use CAI, the time spent always exceeds my original plan.
I often rely on the analysis and suggestions provided by CAI to guide my daily decisions.
I feel irritable when CAI is unavailable.
I feel anxious or worried when CAI is inaccessible.
I feel a sense of loss when I can't access CAI.
I feel helpless and confused when I am unable to use CAI.
I feel like I cannot stop using CAI.
When experiencing negative emotions (such as depression, anxiety, helplessness, guilt, etc.), I tend to use CAI for comfort.
When I feel irritable, I usually use CAI to seek relief.
When I feel lonely, I tend to use CAI to pass the time.
Using CAI effectively helps improve my mood.
People around me often say that I spend too much time using CAI.
After frequently using CAI, I find it more difficult to engage in independent thinking.
Frequent use of CAI has negatively impacted my studies or life.
After using CAI frequently, I have lost interest in other hobbies.
After frequently using CAI, my interactions with friends or family have decreased.
After frequent use of CAI, I feel my ability to engage in active thinking has deteriorated.
