# Correction: Development and validation of the conversational AI dependence scale for Chinese college students

**DOI:** 10.3389/fpsyg.2025.1700281

**Published:** 2025-10-09

**Authors:** Yuanyuan Chen, Mengyun Wang, Shujuan Yuan, Yan Zhao

**Affiliations:** ^1^Department of Psychology, Shaanxi University of Technology, Hanzhong, China; ^2^Department of Psychology, Anhui University of Chinese Medicine, Hefei, China

**Keywords:** conversational artificial intelligence dependence, validation, scale, psychometric, Chinese college students

The Table 1 column headers were in the wrong order in the PDF/HTML version of this paper. The correct order of the column headers should be: Item, Negative impacts, Mood modification, Withdrawal symptoms, Uncontrollability, Communality. The order has now been corrected.

**Table 1:** The four characteristic dimensions of the CAIDS.

**Item**	**Negative impacts**	**Mood modification**	**Withdrawal symptoms**	**Uncontrollability**	**Communality**
33	0.79				0.72
34	0.78				0.78
36	0.74				0.73
47	0.68				0.63
25	0.66				0.67
9	0.65				0.68
11		0.82			0.84
14		0.80			0.77
12		0.79			0.83
15		0.73			0.73
27			0.81		0.83
28			0.78		0.81
30			0.71		0.77
29			0.70		0.75
44			0.59		0.59
4				0.77	0.76
1				0.74	0.74
3				0.73	0.76
5				0.70	0.70
6				0.66	0.69
Eigenvalues	3.95	3.70	3.69	3.53	
Contribution rate	19.75%	18.51%	18.48%	17.67%	
Accumulating contribution rate	19.75%	38.25%	56.73%	74.41%	

*N* = 547.

The original version of this article has been updated.

